# Lichen amyloidosis associated with rheumatoid arthritis: unique presentation in a Bulgarian patient: Amiloidose líquen associada com artrite reumatoide: apresentação única em um paciente búlgaro

**DOI:** 10.1590/1516-3180.2016.024921102016

**Published:** 2017-01-05

**Authors:** Georgi Tchernev, Anastasiya Atanasova Chokoeva, Uwe Wollina

**Affiliations:** I MD, PhD. Professor, Department of Dermatology, Venereology and Dermatological Surgery, Medical Institute of the Ministry of the Interior (MVR-Sofia), Sofia, Bulgaria; Associate Professor, "Onkoderma" Polyclinic for Dermatology and Dermatological Surgery, Sofia, Bulgaria.; II MD. Surgeon, "Onkoderma" Polyclinic for Dermatology and Dermatological Surgery, Sofia, Bulgaria; Chair, Department of Dermatology and Venereology, School of Medicine, Medical University of Plovdiv, Plovdiv, Bulgaria.; III MD, PhD. Director, Department of Dermatology and Allergology, Academic Teaching Hospital Dresden-Friedrichstadt, Friedrichstrasse, Dresden, Germany.

**Figure 1:**
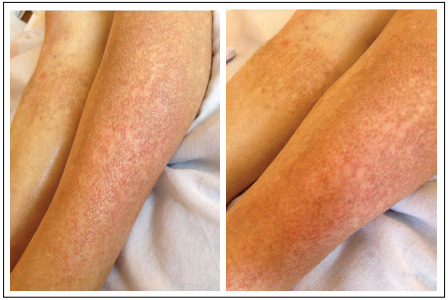
Clinical manifestation of erythematous pruritic papules located on the left pretibial surface of an 80-year-old female patient.

An 80-year-old Caucasian female patient presented with a two-year history of intensively itching skin rash located on her left lower leg and mild swelling of the proximal interphalangeal and metacarpophalangeal joints, accompanied by morning stiffness around these joints, lasting at least one hour before maximal improvement (Figures 1A and 1B). She reported having had a long-lasting medical history of accompanying diseases that had been controlled with medicines. These conditions included arterial hypertension, hypothyroidism, chronic pyelonephritis, angina pectoris and primary glaucoma. There was no family history of cutaneous disorders.

Presence of intensively pruritic erythematous papules located on the left pretibial surface was established clinically (Figures 1A and 1B). Symmetrical soft-tissue swelling around the small joints was also observed, but no rheumatoid nodules were seen. The laboratory blood tests did not reveal any abnormalities in the complete or differentiated blood count. The kidneys and liver showed normal functioning. The rheumatoid factor was 827 u/ml (reference values: less than 40-60 u/ml). Presence of periarticular osteopenia in the interphalangeal and metacarpophalangeal joints was established radiographically. A diagnosis of seropositive rheumatoid arthritis was made, which met most of the criteria postulated by the committee of the American Rheumatism Association.

**Figure 2:**
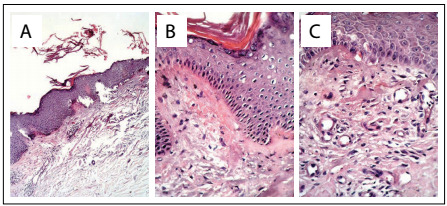
(A) Histopathological findings: focal acanthosis, subepithelial pink-stained structures and mild to moderate mononuclear infiltrate around single vessels (hematoxylin-eosin staining); (B and C) Subepithelial Congo red-positive deposits showing blue-green birefringence under polarized light.

Immunological testing for antinuclear antibody (ANA) and Scl 70 was negative. The cutaneous pathological changes presented required a wide spectrum of differential diagnoses, including pretibial myxedema, necrobiosis lipoidica, the small papular form of cutaneous sarcoidosis, T-cell lymphoma, lichen ruber planus and Arndt-Gottron scleromyxedema. Histopathological evaluations on skin biopsies revealed hyperkeratosis, focal acanthosis, subepithelial structures that stained pink with hematoxylin-eosin and mild to moderate mononuclear infiltrate around single vessels (Figure 2A). Subepithelial Congo red-positive deposits were also observed (Figures 2B and 2C), which showed blue-green birefringence under polarized light.

The findings were characteristic of amyloid deposition and a diagnosis of lichen amyloidosis was made. No clinical or laboratory evidence of systemic amyloidosis was presented. Systemic therapy consisting of bilastine (20 mg daily) and acitretin (15 mg daily) was started, with topical application of 0.1% mometasone furoate cream, which produced a satisfactory therapeutic response. The patient was referred to a rheumatological unit for further therapy with biological agents.

Localized cutaneous amyloidosis encompasses several conditions characterized by deposition of amyloid or amyloid-like proteins in the dermis, including macular amyloidosis and lichen amyloidosis.^1^ Nodular localized cutaneous amyloidosis is another condition in this group: it is the rarest type and distinct from the other two. In this type, plasma cells produce immunoglobulin light chains that are precursors to the amyloid fibril protein called amyloid L.^1^

Lichen amyloidosis is a primary form of localized cutaneous amyloidosis that is clinically manifested through hyperkeratotic erythematous to brownish papules, while amyloid deposition can be seen via specific histological staining in previously normal skin without any evidence of visceral involvement.^2^ The clinical manifestation of these lesions is practically indistinguishable from that of primary and myeloma-associated systemic amyloidosis, and these lesions result from local plasma cell infiltration.^3^

Although cutaneous lesions may be seen in up to 40% of patients with primary and myeloma-associated systemic amyloidosis, their presence results from tissue deposition of immunoglobulin light chain material derived from a circulating paraprotein.^3^ In contrast, amyloid in lichen amyloidosis is not derived from immunoglobulins or serum proteins, but from keratin peptides of necrotic keratinocytes.^4^ Familial predisposition also has a pathogenic role.^2^

Although the etiology is not fully understood, chronic irritation to the skin has been proposed as possible etiological factor.^5^ Chronic scratching is considered to be a cause of damage to keratinocytes in lichen amyloidosis.^2^ The amyloid deposits in patients with lichen amyloidosis are mainly restricted to the upper dermis and arise because of focal epidermal damage with subsequent conversion of necrotic keratinocytes into amyloid in the papillary dermis.^5^ The condition persists for many years with intensive pruritus, but an asymptomatic variant has also been reported in the literature.^6^ ^,^ ^7^

Treatment options include potent topical steroids under occlusion, intralesional steroids, topical dimethylsulfoxide and etretinate.^7^ ^,^ ^8^ Surgical treatment methods include dermabrasion and scalpel scraping of the lesions.^8^ ^,^ ^9^ Given that chronic scratching seems to be the main cause and not the result of the amyloid deposits, treatment should be directed mainly against the pruritus.^4^

We have described a rare association between lichen amyloidosis and rheumatoid arthritis in an 80-year-old female patient, without evidence of systemic amyloid involvement. To the best of our knowledge, this is the first reported case of primary cutaneous amyloidosis in a patient with rheumatoid arthritis, in contrast to the much more frequent association of rheumatoid arthritis with systemic amyloidosis, the pathogenetic relationship remains unclear. It is also unclear whether lichen amyloidosis might be the first clinical manifestation of the initial systemic involvement, in which cutaneous lesions can be seen in up to 40% of the patients,^3^ or whether the pathogenetic relationship of the association is more related to an undefined form of autoimmune dysregulation. Because of the rareness of this simultaneous clinical presentation and limited data in the literature on this issue at this stage, the correct answer to these questions will probably only be given at some point in the future.
